# Clinical and genetic factors associated with kidney tubular dysfunction in a real-life single centre cohort of HIV-positive patients

**DOI:** 10.1186/s12879-017-2497-3

**Published:** 2017-06-05

**Authors:** S. E. Salvaggio, A. Giacomelli, F. S. Falvella, M. L. Oreni, P. Meraviglia, C. Atzori, E. G. I. Clementi, M. Galli, S. Rusconi

**Affiliations:** 1 Infectious Diseases Unit, DIBIC Luigi Sacco - University of Milan, Milan, Italy; 2 ASST Fatebenefratelli-Sacco, Clinical Pharmacology Department, Milan, Italy; 3 ASST Fatebenefratelli-Sacco, First Division of Infectious Diseases, Milan, Italy; 4 Clinical Pharmacology Unit, Consiglio Nazionale delle Ricerche Institute of Neuroscience, DIBIC Luigi Sacco - University of Milan, Milan, Italy; 5 E. Medea Scientific Institute, Bosisio Parini, Italy

**Keywords:** Tenofovir DF, HIV-1, Pharmacogenetics, Tubular dysfunction

## Abstract

**Background:**

Tenofovir (TDF) is one of the most widely used antiretroviral drug. Despite the high degree of tolerability a small percentage of patients experienced alteration in tubular function during TDF use. Intracellular TDF disposition is regulated by ATP-binding cassette (ABC) drug efflux transporters and, a reduced transport activity may be implicated in accumulation of TDF into the cells. The aim of our study was to assess the major determinants of TDF associated tubular dysfunction (KTD) in a real-life setting including the usefulness of single-nucleotide polymorphisms (SNPs) mapping into ABCC2, ABCC4 and ABCC10 genes.

**Methods:**

We retrospectively analyzed all HIV positive patients who were followed at the Infectious Diseases Unit, DIBIC Luigi Sacco, University of Milan from April 2013 to June 2016. All patients treated with TDF who underwent a genotypization for the functional variants mapping in ABCC2 rs717620 (−24 C > T), ABCC4 rs1751034 (3463 A > G) and ABCC10 rs2125739 (T > C) were evaluated. KTD was defined as the presence of urine phosphate wasting and/or proteinuria at 24 h urine analysis.

**Results:**

One hundred fifty-eight patients were genotyped, of which 42 (26.6%) experienced signs of KTD. No statistical significant differences were observed among patients with or without KTD regarding age, gender, ethnicity and comorbidities (hypertension and diabetes). The percentage of patients with KTD was higher among those with “GG” genotype at rs1751034 of ABCC4 compared to patients without KTD [6 (14.3%) vs 4 (3.5%), *p = 0.01*]. No statistical significant differences were observed regarding the distribution of ABCC2 and ABCC10 SNPs. Carriers of “G” allele in homozygous status at rs1751034 of ABCC4 showed a significant association with KTD (Odds Ratio 4.67, 95% CI 1.25–17.46, *p = 0.02*) in bivariate analysis, but this association was lost in multivariable analysis. A significant association between bone diseases and KTD was observed (Odds Ratio 3.178, 95%CI 1.529–6.603, *p = 0.002*).

**Conclusions:**

According to our results ABCC4 rs1751034 could be a genetic determinant of KTD; however validation studies are needed for therapy personalization. Noteworthy, a strong association between bone disease and KTD was also observed.

## Background

Tenofovir disoproxil fumarate (TDF), a prodrug of Tenofovir, is a nucleotide reverse-transcriptase inhibitor used for the treatment of human immunodeficiency virus type 1 (HIV-1) infection. It is widely used because of its high potency, good safety profile, convenient once daily dosing and relatively minimal adverse reactions [1]. After absorption, TDF is converted to tenofovir that then is phosphorylated intracellularly to tenofovir diphosphate, an active analog, which inhibits HIV reverse transcriptase, arresting the elongation of DNA chain. Tenofovir is eliminated by renal excretion through glomerular filtration and active tubular secretion. Although tenofovir causes a mild degree of nephrotoxicity, several cases of Fanconi syndrome and acute renal failure have been reported in patients on this therapy [2, 3]. The mechanism of tenofovir-induced kidney injury isn’t completely understood. Mitochondrial damage in proximal renal tubular cells is observed in patients with significant tenofovir induced kidney tubular dysfunction (KTD) [4]. Furthermore, because characteristics and severity of KTD vary among individuals, the role of host genetics seems to be influential. Tenofovir is secreted into tubular lumen by energy-dependent pumps, Multidrug-Resistance Proteins 2 (MRP2), MRP4 and MRP7 encoded by genes ABCC2, ABCC4 and ABCC10, respectively [5, 6]. Therefore, genetic variants affecting these transport activity may be implicated in tenofovir-accumulation into the cells and in tenofovir-KTD. Previous studies have shown that some Single-Nucleotide Polymorphism (SNPs) into ABCC2, ABCC4 and ABCC10 genes are associated with higher tenofovir plasma and/or intracellular levels [7–10]. More in details, for ABCC2 rs717620 at position −24 (−24C > T), individual with KDT was significantly higher in CC than CT/TT carriers and, a correlation between ABCC2 CC (−24C > T) and a higher plasma tenofovir concentration was also reported [6, 11]. A study by Kiser et al. observed that patients carrying the ABCC4 3463A➔G variation had lower renal clearance of tenofovir than those without. Finally, two SNPs mapping in ABCC10 gene (rs9349256 and rs2125739) and their haplotype were significantly associated with KTD [10].

The aim of this study was to investigate the major determinants of TDF associated tubular dysfunction (KTD) including the correlation between polymorphisms in ABCC2, ABCC4 and ABCC10 genes and the tenofovir-induced KTD in a mono-centric cohort of HIV-1 positive patients.

## Methods

We performed a single centre retrospective observational cohort study in the cohort of patients followed at the University Infectious Disease Unit of Hospital Luigi Sacco in Milan from April 2013 to June 2016. All patients who had been treated with a TDF-containing antiretroviral regimen and were referred to our out-patient clinic for the management and prevention of antiretroviral toxicities underwent, as part of clinical practice assessment, a genotyping for ABCC2 rs717620 (−24 C > T), ABCC4 rs1751034 (3463 A > G) and ABCC10 rs2125739 (T > C). Genomic DNA was isolated from peripheral blood cells using an automatic DNA extraction system (Maxwell® 16 System, Promega) according to the manufacturer’s instructions. All genotypes were determined by Real-Time PCR, using a panel of LightSNiP from TIB-MolBiol (assays based on SimpleProbe®). At the end of the amplification a melting curve analysis was performed (LightCycler 480, Roche). All data were collected in an electronic database. We collected data on gender, epidemiology, race, immuno-virological situation at the start of TDF, antiretroviral history, comorbidities (bone, cardiovascular, diabetes) and urine examination comprehensive of a 24 h sample. We defined KTD in our cohort as the presence of abnormal proteinuria (24 h urine proteins >150 mg) and/or phosphaturia (24 h urine phosphate >1200 mg) at 24 h urine collected during the period of treatment with TDF [12, 13]. The definition of osteopenia and osteoporosis were based on DEXA scan assessment: bone mineral density Z-score between −1 and −2.5 standard deviation (SD) and below −2.5 SD, respectively. Hypertension was defined as having a blood pressure > 140/90 mmHg in two consecutive assessments or if the patient was on antihypertensive drugs. Diabetes was defined as having a fasting glucose level > 126 mg/dL in two consecutive determination or if the patient was taking antidiabetic drugs.

The aim of our study was to assess the distribution of the different ABCC2, ABCC4 and ABCC10 genotypes in patients treated with TDF with or without KTD. Baseline characteristics were compared between patients with or without KTD by using a descriptive statistical analysis (χ2 or *Fisher’s exact test* for categorical variables and Mann-Whitney test for continuous variables). Associations between genotypes and KTD were tested by univariate and multivariate logistic regression analyses. The impact of all variables was estimated with univariate analysis, and those with *P* value < 0.20 were incorporated into multivariate analysis. Statistical significance was defined at 2-sided *P* value < 0.05. To perform statistical analysis we used the SAS software version 9.3.

## Results

### Baseline characteristics of patients

One hundred fifty-eight patients were evaluated over a median follow-up time of 66 months (Inter quartile range [IQR] 24–101), whose characteristics are shown in Table 1. During the period of observation, KTD was observed in 42 (26.6%) patients. No statistically significant differences were observed between patients with and without KTD regarding age, gender and ethnicity [non-Caucasian patients were 2 (4.8%) in the KTD group and 3 (2.6%) in patients without KTD *p* = 0.490]. Patients with KTD have a longer previous duration of therapy when compared with patients without KTD [0,6 years (IQR 0.0–8.29) vs 0,0 years (IQR 0.0–3.8); *p = 0.034*], accordingly KTD was present in a lower number of patients who received TDF as part of their initial antiretroviral regimen [14 (33,3%) vs 69 (59,5%); *p = 0,004*]. Furthermore, the duration of the TDF-containing regimen appeared to be longer in patients with KTD compared to that in patients without KTD although not statistically significant [76 months (IQR 30–110) vs 59 months (IQR 23–95); *p = 0.090*]. In both groups, kidney function at baseline, evaluated through creatinine and GFR values, and body mass index were similar. About baseline comorbidities no differences were observed between the two groups regarding hypertension ad diabetes, while a higher prevalence of bone disease (osteopenia/osteoporosis) in patients with KTD [23 patients (54.8%) vs 32 patients (27.6%); *p = 0.002*] was observed.

**Table 1:** Characteristics of the study population

	Overall population *N* = 158		Patients with KTD *n* = 42		Patients without KTD*n* = 116		p*
Female (%)	34	21.5%	8	19.0%	26	22.4%	0.649
Age [yrs] median (IQR)	42	35–48	42	37–45	42	34–49	0.591
Non-Caucasian (%)	5	3.2%	2	4.8%	3	2.6%	0.490
BMI (Kg/m^2^) median (IQR)	22,9	20,3–25,2	23,5	20,7–25,4	22,5	20,2–25,2	0.339
TDF as part of first ARV regimen (%)	83	52,5%	14	33,3%	69	59,5%	*0.004*
Previous therapy duration [yrs] median (IQR)	0.0	0.0–6.1	0.6	0.0–8.2	0.0	0.0–3.8	*0.034*
TDF duration (months) median (IQR)	66	24–101	76	30–110	59	23–95	0.090
Use of protease inhibitors (%)	103	65.2%	26	61.9%	77	66.4%	0.602
CD4+ (cells/μL) median (IQR)	373	228–599	430	251–635	371	224–580	0.638
HIV-RNA (log cps/mL) median (IQR)	3.8	0.0–5.0	0.0	0.0–4.5	4.0	0.0–5.0	*0.012*
Creatinine (mg/dL) median (IQR)	0.84	0.72–0.94	0.84	0.70–0.99	0.83	0.73–0.92	0.534
GFR (MDRD equation) median (IQR)	103	89–117	106	89–118	101	89–117	0.586
HCV coinfection (%)	18	11.4%	6	14.3%	12	10.3%	0.491
Diabetes (%)	11	7.0%	5	11.9%	6	5.2%	0.142
Hypertension (%)	20	12.7%	8	19.0%	12	10.3%	0.146
Bone disease^a^ (%)	55	34.8%	23	54.8%	32	27.6%	*0.002*

*Abbreviations*: *yrs.* years, *IQR* Inter quartile range, *cps* copies, *BMI* body mass index, *GFR* glomerular filtration rate, *MDRD* The Modification of Diet in Renal Disease, *TDF* tenofovir diproxil fumarate, *ARV* antiretroviral therapy; ^a^osteopenia/osteoporosis; *p values are for χ2 or Fisher’s exact test and Mann-Whitney test

### Genotype distribution and factors associated with KTD

**Fig. 1:**
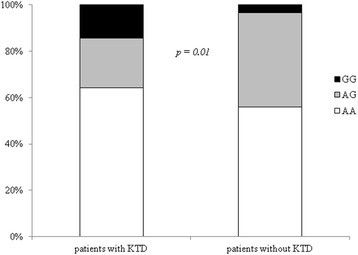
Distribution of different genotypes at rs1751034 of ABCC4 gene in patients with or without KTD. Abbreviations: KTD = kidney tubular disfunction; *χ2

The distribution of the polymorphism for ABCC2, ABCC4 and ABCC10 are shown in Table 2. A significant statistical difference was observed in the distribution of the variant in rs1751034 of ABCC4 in patients with KTD when compared with patients without KTD. In particular the “GG” genotype at the position 3463 was more prevalent in patients with KTD [6 (14,3%) vs 4 (3.5%); *p = 0.01*] (Fig. 1). No statistical significant differences were observed among the ABCC2 rs 717,620 (−24 C > T) and ABCC10 rs2125739 (T > C) genotypes. As shown in Table 3, univariate analysis shows a significant association between KTD and the patients with the genotype GG at the position 3463 of ABCC4 [Odds ratio (OR) 4.667 (IC 95% 1.247–17.464); *p = 0.022*]. When we considered clinical parameters a significant association between bone diseases and KTD was observed [OR 3.178 (IC95% 1.529–6.603); *p = 0.002*], while an inverse correlation was observed between baseline HIV-RNA [OR 0.793 (IC 95% 0.677–0.929); *p = 0.004*] and receiving TDF as part of the initial antiretroviral regimen [OR 0,341 (IC 95% 0.162–0.715); *p = 0.004*]. In the multivariable model the association with the GG genotype was lost [OR 2.663 (IC95% 0.610–11.621); *p* = 0.193] whereas the association with bone disease maintained a statistical significance [OR 3.147 (IC95% 1.361–7.279); *p = 0.007*]. A trend between diabetes and KTD emerged [OR 3.670 (IC 95% 0.914–14.740); *p* = 0.067] in parallel.

**Table 2:** Distribution of different genotypes at ABCC2 rs 717,620 (−24 C > T), ABCC4 rs1751034 (3463 A > G) and ABCC10 rs2125739 (T > C) in patients treated with tenofovir with or without KTD

	Overall population *N* = 158		Patients with KTD *n* = 42		Patients without KTD *n* = 116		p*
ABCC2 -24 C > T, rs717620 (%)							
C/C	104	65.8%	25	59.5%	79	68.1%	0.503
C/T	52	32.9%	16	38.1%	36	31.0%	
T/T	2	1.3%	1	2.4%	1	0.9%	
ABCC4 3463 A > G, rs1751034 (%)							
A/A	92	58.2%	27	64.3%	65	56.0%	*0.010*
A/G	56	35.5%	9	21.4%	47	40.5%	
G/G	10	6.3%	6	14.3%	4	3.5%	
ABCC10 rs2125739, T > C (%)							
T/T	80	50.6%	19	45.2%	61	52.6%	0.394
T/C	66	41.8%	21	50.0%	45	38.8%	
C/C	12	7.6%	2	4.8%	10	8.6%	

*Abbreviations*: *KTD* kidney tubular dysfunction, *ABCC* ATP-binding cassette; *χ2

**Table 3:** Logistic regression of factors involved in the development of KTD

	Bivariate analysis				Multivariate analysis			
	OR	(95% CI)		*p*	OR	(95% CI)		*p*
Female vs male	0,814	0,336	1974	0,650				
Caucasian vs Non-Caucasian	1883	0,304	11,684	0,497				
Baseline age (per yrs)	0,989	0,956	1023	0,524				
BMI ≤18 vs > 18	1133	0,335	3840	0,841				
TDF as part of first ARV regimen vs TDF as part of second or further ARV regimens	0,341	0,162	0,715	*0,004*	1040	0,195	5537	0,963
Previous therapy duration (per yrs)	1057	0,988	1131	0,110	0,948	0,854	1051	0,309
Baseline CD4 cell count (per cells/mL)	1000	0,999	1001	0,907				
Baseline Plasma HIV-RNA level (per log cps/mL)	0,793	0,677	0,929	*0,004*	0,809	0,578	1132	0,216
HCV coinfection	1444	0,505	4131	0,493				
Baseline creatinine (per mg/dL)	1999	0,138	28,861	0,611				
Duration of treatment with TDF (per months)	1006	0,998	1015	0,162	0,998	0,987	1009	0,672
Use of protease inhibitors	0,823	0,396	1712	0,602				
Hypertension	2039	0,769	5405	0,152	0,991	0,318	3084	0,987
Diabetes	2477	0,714	8595	0,153	3670	0,914	14,740	0,067
Bone disease^a^	3178	1529	6603	*0,002*	3147	1361	7279	*0,007*
ABCC2–24 C > T (CC vs CT/TT)	0,689	0,332	1428	0,316				
ABCC4 3463 A > G (GG vs AG/AA)	4667	1247	17,464	*0,022*	2663	0,610	11,621	0,193
ABCC10 rs2125739 T > C (CC vs TC/TT)	0,530	0,111	2525	0,425				

*Abbreviations*: *OR* odds ratio, *CI* confidence interval, *cps* copies, *yrs.* years, *BMI* body mass index, *KTD* kidney tubular dysfunction, *ABCC* ATP-binding cassette, *ARV* antiretroviral therapy; ^a^osteopenia/osteoporosis; *TDF* tenofovir diproxil fumarate; *p* values are for χ2

## Discussion

We performed our study in a population setting of HIV-1 positive patients treated with TDF. The present study showed an increased prevalence of the variant rs1751034 of ABCC4 in HIV-1 positive patients treated with TDF manifesting signs of KTD; in particular an association with the risk of KTD and ABCC4 genotype “GG” was shown in bivariate analysis despite we could not confirm this finding in multivariable analysis. In vitro studies demonstrated the involvement of ABCC4 in the luminal efflux of adefovir and TDF in the kidney, thus justifying a decrease in the tubular drug secretion and an intracellular accumulation of these drugs [14–16]. The association with polymorphisms of ABCC4, in particular 4976, and the presence of beta2 microglobulinuria in Thai HIV-1 positive patients was previously described by Likanonsakul et al. In the same population approximately 20% of patients manifested signs of KTD with a median of 5.04 years on TDF (IQR 3.9–6.7) [7]. This findings are similar with the prevalence of KTD observed in our cohort which has also a comparable previous period on TDF [5.5 years (IQR 2.0–8.5)]. In another study the same polymorphism was associated with a reduced exclusion of TDF, despite the plasmatic concentration of the drugs didn’t differed between different genotypes at ABCC4 4976 [17]. In a previous study by Rodríguez-Nóvoa et al. an association with the ABCC 4669 genotype CC and phosphorus wasting was observed [6]. This observation could partially explain our findings due to the fact that we used phosphate wasting as a criteria to discriminate between patients with and without KTD. In a study in Japanese HIV positive patients no associations was observed between polymorphisms of ABCC4 and the risk of KTD, in the same study a strong association between the genotype CC at position −24 of ABCC2 gene was observed. We couldn’t confirm these findings in our real life patients setting [9]. In another large study performed by Nishijima et al. in the same setting no association was observed between ABCC2 (−24C > T) and major renal outcome (>10 ml/min/1.73 m2 decrement in eGFR relative to baseline, >25% decrement in eGFR, and eGFR <60 ml/min/1.73 m2) related to TDF toxicities and the authors concluded that such SNPs are not to be considered a risk factor for clinical TDF-related renal dysfunction [18]. Similar findings were also reported in another study in which ABCC2 haplotypes was associated with renal proximal tubulopathy induced by TDF in HIV-1-infected patients, but no association was observed between ABCC4 polymorphism [19]. Despite several report of association of polymorphisms of ABCC2 and the development of KTD in patients treated with TDF it is notable that the exact mechanism by which these SNPs poses at risk of KTD development remains unknown. In particular MPR2 encoded by ABCC2 doesn’t seems to be involved in the luminal transport of TDF in the tubular cells of the kidney [14]. In an in vitro study TDF renal tubular transport seems to be influenced by genetic variation of ABCC10, the authors suggested a possible implication in ABCC10 variations and the development of ABCC10 [10]. We weren’t able to confirm the association of ABCC10 rs2125739 and KTD in our cohort. In our study, traditional risk factor for KTD like diabetes or hypertension are equally distributed between the patients with and without KTD with a general prevalence of 7% of diabetes and 12% of hypertension. In multivariable analysis a trend was observed with diabetes posing patients at greater risk of developing KTD. It’s important to manage this modifiable classic risk factors for kidney damage especially when no clear-cut results are available from basic research and a lacking association remain with SNPs and KTD [20]. We observed a significantly higher prevalence of bone disease in patients with signs of KTD when compared with patients without KTD (54,8 vs 27,6%; *p = 0.002*) and a strong association between bone disease an KTD was also confirmed in the multivariable analysis. As previously described in HIV positive patients treated with TDF a strong association was observed between KTD and a lower bone mineral density [21, 22]. In addition the prevalence of bone disease (osteopenia/osteoporosis) such as the prevalence of KTD was similar to previous report by Calmy et al. [23].

Our study has several limitations, in particular the retrospective design of the study exposed the analysis to biases due to the lack of data that hadn’t been reported. The single centre design reduce the applicability of our findings to other cohorts, e.g. we have a very little presence in this study of non-Caucasian patients. Due to the limited size of our sample, we cannot rule out that the association of the genotype GG at ABCC4 3463 with tubulopathy could be driven by chance. The high prevalence of bone disease in our population could suggest also a channelling bias due to the selection of patients picked out in clinical practice – roughly one third of patients who had been taking TDF – for genotypization due to the concomitant presence of comorbidities. The criteria used to define KTD were also subjected to limitation due to the potential intervention of confounders and lack of specificity for proximal tubular damage.

## Conclusions

We observed an association between the variant rs 1,751,034 of ABCC4 and KTD in our single centre cohort of HIV patients treated with TDF. A strong association between bone disease and KTD was also observed. This finding should prompt our attention on the parallel onset of these 2 co-morbidities and the consequent effort to manage affected subjects in a timely manner.

## Abbreviations

ABC: ATP-binding cassette
HIV-1: Human immunodeficiency virus type 1
IQR: Inter quartile range
KTD: Kidney tubular dysfunction
MRP: Multidrug-Resistance Proteins
SD: Standard deviation
SNPs: Single-Nucleotide Polymorphism
TDF: Tenofovir disoproxil fumarate
